# Orthostatic Hypotension in Benign Prostatic Hyperplasia Patients and Its Association With Alpha-1 Antagonist Use: A Comprehensive Literature Review

**DOI:** 10.7759/cureus.44097

**Published:** 2023-08-25

**Authors:** Muhammad Abubakar, Rachna Prasad, Siffat S Salim, Deepak Basavaraju, Munazza Khan, Ibrahim K Lateef, Ahmad Furqan, Saud Raza, Ishita Gupta, Deepak Singla, Hira Adil, Ather Naeem

**Affiliations:** 1 Department of Internal Medicine, Siddique Sadiq Memorial Trust Hospital, Gujranwala, PAK; 2 Department of Internal Medicine, Ameer-ud-Din Medical College, Lahore General Hospital, Lahore, PAK; 3 Department of Internal Medicine, Stanley Medical College, Chennai, IND; 4 Department of Surgery, Holy Family Red Crescent Medical College Hospital, Dhaka, BGD; 5 Department of Internal Medicine, Mysore Medical College and Research Institute, Mysore, IND; 6 Department of Internal Medicine, Medical University Pleven, Pleven, BGR; 7 Department of Internal Medicine, Lahore Medical and Dental College, Lahore, PAK; 8 Department of Internal Medicine, Dr. Baba Saheb Ambedkar Medical College and Hospital, New Delhi, IND; 9 Department of Internal Medicine, Government Medical College, Patiala, Patiala, IND; 10 Department of Community Medicine, Khyber Girls Medical College, Peshawar, PAK; 11 Department of Cardiology, Punjab Institute of Cardiology, Lahore, PAK

**Keywords:** quality of life, orthostatic hypotension, lower urinary tract symptoms, benign prostatic hyperplasia, alpha-1 adrenergic antagonists

## Abstract

Orthostatic hypotension (OH) is frequently observed in benign prostatic hyperplasia (BPH) patients undergoing alpha-1 adrenergic antagonist (A1AA) therapy. While previous studies have acknowledged the prevalence of OH in BPH patients on A1AAs, limited data exist on ranking the safety of different A1AAs. This comprehensive review explores the underlying mechanisms of OH, examines numerous factors influencing its development, and provides insights into effective treatment strategies such as hydration, gradual postural changes, leg exercises, compression stockings, and tilt-table training for BPH management. The review highlights the significance of individualized care, interdisciplinary collaboration, and further research to optimize A1AA treatment, improve patient outcomes, and enhance quality of life.

## Introduction and background

Among aging men, benign prostatic hyperplasia (BPH) is a prevailing condition that can significantly affect their quality of life (QoL) due to bothersome symptoms of the lower urinary tract (LUTS). It is marked by the gradually increased prostate size due to the benign proliferation of epithelial and smooth muscle (SM) cells [1]. Tamsulosin and alfuzosin, which belong to alpha-1 adrenergic antagonists (A1AA), are effective treatment options that reduce LUTS and improve urine flow rates [2]. However, the relationship between the usage of A1AA and orthostatic hypotension (OH) in BPH patients remains an important topic of investigation.

OH is characterized by a notable decline in BP when transitioning to an upright posture. This condition is identified by at least a 20 mmHg drop in systolic BP, at least a 10 mmHg in diastolic BP, or both, within 3 minutes upon standing [3,4]. This condition can lead to various symptoms and complications, affecting the QoL and functional independence of affected individuals [3]. Understanding the incidence and prevalence of OH in BPH patients taking A1AAs is crucial for optimizing treatment strategies and minimizing potential risks.

Despite extensive research on both BPH and OH individually, a literature gap lies in the comprehensive understanding of the incidence and prevalence of OH in BPH patients prescribed A1AAs. Studies have explored the association between A1AA use and OH. Still, there remains a need for a comprehensive literature review to consolidate existing evidence, identify research gaps, and provide insights for further investigation.

Furthermore, the specific role of A1AAs in causing or exacerbating orthostatic symptoms in BPH patients remains unclear. Although these medications target alpha-1 adrenergic receptors (A1AR) in the bladder-neck and prostate gland, they may also affect A1ARs in blood vessels, potentially contributing to BP dysregulation [5]. Therefore, addressing this gap and clarifying the unknown aspects of A1AAs' involvement in orthostatic symptoms among BPH patients is essential. This comprehensive literature review aims to bridge these gaps in the existing knowledge by examining the occurrence, frequency, and specific role of A1AAs in OH among patients with BPH.

## Review

Methodology and article selection

Electronic searches were conducted on the following databases: PubMed, PubMed Central, and Google Scholar to compile relevant articles. The search was conducted from the inception of these databases until August 3, 2023. A Boolean search approach was employed, incorporating medical subject headings (MeSH), regular keywords, and synonyms pertinent to the subject. These included "Orthostatic Hypotension," "Benign Prostatic Hyperplasia," and "Alpha-1 Adrenergic Antagonist," both individually and in combination. The search process was optimized using the search functions provided by the websites. Articles of various types were taken into consideration. After the search process, 178 articles were retrieved and organized. After the initial review of titles and abstracts, 127 articles were excluded. Additionally, the search encompassed the exploration of grey literature that added 16 articles. The final compilation comprises 67 articles, chosen based on their relevance to the topics deliberated in this review.

Overview of OH

Pathophysiology and Underlying Mechanisms of OH - Autonomic Dysfunction, Impaired Baroreflex Response, and Hemodynamic Changes

OH can arise from a range of causative factors, encompassing both neurologic and non-neurologic origins, as well as from the effects of certain medications. Neurogenic OH arises from dysregulation of the autonomic nervous system, linked to neuropathic diseases, neurodegenerative diseases, or because of the aging process. Neuropathic conditions encompass diabetes, familial dysautonomia, and autoantibodies against cholinergic receptors. Neurodegenerative diseases include Parkinson's disease, multiple system atrophy, and pure autonomic failure. In contrast, non-neurogenic OH commonly arises from diminished fluid volume [6]. Furthermore, medication-induced OH should be considered, especially in older individuals on multiple medications [7].

Two types of responses can occur in OH: "classic" OH, which occurs within three minutes of standing, and "delayed" OH, which manifests after 3 minutes. While extensive research has focused on classic OH, less is known about the delayed variant of this condition [8]. Further investigations are warranted to enhance our understanding of its underlying mechanisms and clinical implications.

When individuals transition from a lying down position to standing up, blood tends to accumulate in the legs due to the gravitational pull. This pooled blood, ranging from 300 to 800 mL, leads to a decreased venous return, preload, and stroke volume, ultimately resulting in a reduction in cardiac output, as observed on the Frank-Starling Curve. The body typically initiates a response known as the baroreceptor reflex to counterbalance this Frank-Starling effect. This reflex involves an increase in sympathetic tone, which causes a rise in peripheral vascular resistance. Consequently, cardiac output and venous return are augmented, thereby mitigating the decline in BP [9]. However, individuals with an inadequate compensatory mechanism are prone to experiencing symptoms associated with OH.

Symptoms, Signs, and Complications Associated With OH

OH presents with clinical symptoms, including impaired vision, fatigue, lethargy, lightheadedness, and cognitive dysfunction. Cognitive impairment and decreased cerebral perfusion may contribute to difficulties in concentration, memory, and overall cognitive function. In more severe instances, syncope (fainting) could manifest, increasing the risk of falls and injuries. OH-related falls are a significant concern, especially in older individuals, as they can lead to fractures, trauma, and reduced mobility [10].

A1AA in BPH treatment

Overview of A1AA Commonly Used for BPH - Tamsulosin, Alfuzosin, and Silodosin

The three subtypes of A1ARs, A1aARs, A1bARs, and A1dARs, can be found in various tissues, especially the LUTS and blood vessels. Among these subtypes, A1aARs and A1dARs play crucial roles in regulating SM contractility and BP, particularly in blood vessels [11]. On the other hand, the exact function of A1bARs in SM contractility and BP control still needs to be fully understood [12].

A1AA - The Ability to Relax Smooth Muscle in the Bladder-Neck and the Prostate

The mechanism of action of A1AAs in BPH treatment centers around their ability to selectively block A1ARs. SM cells in the bladder-neck and prostate (A1aARs), along with arterioles (A1bARs), exhibit a substantial abundance of these receptors [13]. By binding to these receptors, A1AAs prevent the action of norepinephrine, a neurotransmitter that promotes SM contraction. As a result, these SMs relax and reduce urinary obstruction, facilitating urine flow [14].

Comparative Efficacy and Selectivity of A1AA in Relieving LUTS - Improving Urine Flow Rate and Reducing Post-Void Residual Volume (PRV)

Multiple studies have indicated the effectiveness of A1AAs in alleviating LUTS and improving urine flow rate [14]. Furthermore, A1AAs have also been found to decrease PRV, indicating their effectiveness in improving bladder emptying [15]. It is important to note that a wide range of A1AAs are available, and they differ in their selectivity for A1a and A1d receptor subtypes. This selectivity may influence their side effect profile, including the potential risk of OH. Medications with higher selectivity for the A1aAR, such as tamsulosin, have been associated with a reduced likelihood of OH than those with broader receptor selectivity [16].

Among the A1AAs commonly prescribed for BPH are tamsulosin, alfuzosin, and silodosin. These medications have proven efficacy in relieving LUTS and improving urinary function [16,17].

Tamsulosin exhibits a high selectivity for the A1aAR subtype. By blocking these receptors, tamsulosin effectively relaxes the SMs, alleviating urinary obstruction and promoting better urine flow [18]. Clinical studies have consistently shown the efficacy of tamsulosin in improving LUTS, including reduced urinary frequency, enhanced urine flow rate, and decreased PRV [19]. Furthermore, OH is relatively lower with tamsulosin than with similar A1AAs, making tamsulosin a preferred choice for patients at risk of BP fluctuations [16].

In patients with BPH, alfuzosin, a non-selective A1AA, has been found to be more concentrated in the prostate than in the plasma despite targeting all three receptor subtypes. Its selectivity is attributed to its preferential localization at the urinary level that allows it to cause effective relaxation of the SM in both the bladder-neck and prostate [20]. Three clinical trials evaluating the pharmacodynamic profile of alfuzosin have been conducted and revealed that a daily formulation of 10 mg alfuzosin over three months has high tolerability and minimal vasodilatory effects compared to older alpha-blocking compounds [21]. Additionally, alfuzosin has a lower incidence of ejaculation disorders, further highlighting its favorable side effect profile [22]. Clinical research has confirmed the efficacy of alfuzosin in relieving LUTS, with patients reporting improvements in urine flow, reduced urgency, and decreased PRV [23,24]. It is important to note that alfuzosin, like other A1AAs, may cause OH as a potential side effect. However, the risk of OH with alfuzosin is relatively low, particularly compared to non-selective A1AAs [20].

Silodosin is a newer A1AA that has gained recognition for its effectiveness in reducing LUTS. Like tamsulosin, silodosin exhibits a high selectivity for the A1aAR subtype. However, its selectivity is higher than tamsulosin. This selectivity allows targeted SM relaxation in the prostate, improving urine flow [25]. Clinical trials have demonstrated the efficacy of silodosin in relieving LUTS. When administered at an 8 mg daily dose, silodosin demonstrated notable benefits in patients participating in clinical studies. Compared with those who received a placebo, these men experienced substantial improvements in the International Prostate Symptom Score (IPSS) and maximal urine flow rate. The efficacy of silodosin was evident in addressing both voiding and storage symptoms associated with BPH [26]. Studies have shown that silodosin does not worsen OH, with only a minute fraction of patients discontinuing the medication due to this side effect. Similarly, while ejaculatory dysfunction was a significantly reported adverse effect, it had a low discontinuation rate due to the minimal bothersome symptoms in this age group. These findings support silodosin as a favorable option for managing LUTS in older adults taking antihypertensive drugs, offering improvements in urinary symptoms without exacerbating OH [27].

Impact of Different Dosages and Treatment Durations of A1AA on the Incidence and Prevalence of OH in BPH Patients

While all A1AAs demonstrate similar efficacy in treating LUTS associated with BPH, it should be noted that the incidence of OH differs with different A1AAs [16]. Among them, the extended-release (ER) formulations of alfuzosin and tamsulosin demonstrate the lowest tendency to cause adverse effects related to lowering BP [16].

Among the first-line drugs, tamsulosin, which has 20-38-fold greater affinity for A1aAR than for A1bAR, is known to be safer than other A1AAs in regard to causing OH, according to several studies [15]. The incidence of OH in patients taking tamsulosin is 42 events per 10,000 person-years [28]. Tamsulosin can be taken once a day and is preferred as there is no requirement for dose adjustment of antihypertensive drugs like nifedipine, enalapril, or atenolol. In elderly patients and those with cardiovascular comorbidities on concurrent medications, tamsulosin is tolerated well since it has a modest impact on BP [29,30]. Notably, tamsulosin 0.4 mg has the least likelihood of reducing BP and is associated with fewer symptomatic OH instances than terazosin [30]. Furthermore, tamsulosin up to 0.8 mg is also proven safe to use without increased risk of OH [31].

Conversely, alfuzosin has a more dramatic influence on BP than tamsulosin, particularly in older individuals. Due to unfavorable vasodilatory effects, it is frequently discontinued in individuals over 75 with LUTS who simultaneously receive therapy for concomitant cardiovascular conditions [16]. Overall, alfuzosin offers a favorable safety profile, with a minimal risk of adverse effects at the initial dosage [32].

Silodosin is associated with few dizziness events and has a low incidence of OH. Its rapid onset of clinical effectiveness makes it a valuable choice for treating patients with BPH [33]. Moreover, a comparative study found that the occurrence of OH with silodosin was on par with that reported for tamsulosin. The study also found that silodosin improves subjective symptoms in initial and crossover therapy, improving the QoL in individuals with LUTS [26].

Risk factors for OH in BPH patients

The development of OH may not solely be attributed to using A1AAs, as various patient characteristics and comorbidities also play a significant role.

Risk Factors Associated With OH in BPH Patients Taking A1AA

The plasma volume decreases by 10-15 percent upon standing due to venous pooling; the body responds to this through a tightly regulated system involving baroreceptors and veno-arterial stretch receptors through autonomic nervous system (ANS) neural control mechanisms. Normal aging associated with autonomic dysfunction and vascular stiffening associated with hypertension impairs normal vascular responsiveness [34]. As BPH often presents in older people, who most often also have cardiovascular comorbidities and autonomic dysfunction, we must consider the risk of adverse effects like OH in such a patient population [29].

Age-Related Changes in Autonomic Function and Vascular Responsiveness as Important Risk Factors

Age-related alterations in autonomic pathways, degeneration of carotid baroreceptors, and vascular changes establish an autonomous connection between aging and OH [35]. First-line treatments for BPH, like A1AAs, are also known to affect vascular tone through A1bARs and thus may have an additive effect in causing OH [36]. Deconditioning associated with immobility and the predisposition of older people to atrial fibrillation also contribute to OH in this age group [34]. OH is also strongly related to decreased cerebral blood flow [10].

Comorbidities and Increased Susceptibility to OH

Patients with underlying cardiovascular conditions, including hypertension and coronary artery disease, may already have compromised cardiovascular function. The concurrent use of A1AAs, which exert vasodilatory effects, can further potentiate the risk of OH in these individuals [37]. Additionally, conditions such as autonomic neuropathy and diabetes mellitus, which affect autonomic nervous system function, can predispose BPH patients to OH. Efferent sympathetic vasomotor denervation significantly impacts the occurrence of OH in type 2 diabetes mellitus, leading to reduced vasoconstriction in the splanchnic and other peripheral vascular beds [38].

Potential Interaction Between A1AA and Other Medications That Can Contribute to OH - Antihypertensives, Diuretics, and Psychotropic Drugs

Elderly patients often take multiple medications known to induce OH due to drug interactions. Interactions between A1AAs, diuretics (inducing hypovolemia), and psychotropic medications (with A1AA properties) have been well described. A1AAs, antidepressants, antipsychotics, benzodiazepines, beta-adrenergic antagonists, calcium channel blockers, nitrates, and opioids cause OH independently, but their underlying mechanism remains unclear [39]. Thus, polypharmacy poses a notable risk factor in BPH patients. Withdrawal of antihypertensives has also been known to cause this side effect.

Clinical implications and management strategies

Non-pharmacological Approaches

Implementing non-pharmacological approaches for managing OH requires educating patients about the possible risks and the significance of postural changes, promoting hydration, encouraging gradual posture adjustments, advising against prolonged sitting or standing, emphasizing regular exercise, suggesting pre-standing leg exercises, recommending compression garments and considering supervised tilt-table training as part of a comprehensive management plan.

Educating patients about the potential risk of OH and the importance of postural changes: It is crucial to educate patients about the risks of OH and stress the importance of proper postural adjustments, as detailed in Table 1.

**Table 1:** Strategies for Management of Two Common Subtypes of Orthostatic Hypotension This table provides comprehensive strategies for managing different aspects of orthostatic hypotension, including early morning challenges, postprandial concerns, and nighttime adjustments. OH: orthostatic hypotension, BP: blood pressure

Category	Instructions
Early Morning OH	Elevate the head of the bed to reduce nocturia and promote better BP control; exercise caution when waking up; gradually shift from a supine to an erect position to allow the body to adjust; and, drink two cups of cold water approximately 30 minutes before getting up to help regulate BP.
Postprandial OH	Understand that patients with diabetic neuropathy are more prone to experiencing postprandial OH; be mindful that hot drinks and carbohydrate-rich foods may exacerbate symptoms; and, take short, frequent meals.

Adequate hydration to maintain blood volume: Dehydration can exacerbate OH by diminishing blood volume. A comprehensive analysis was conducted to assess the beneficial effects and safety of combining A1AAs with anticholinergic agents (a cause of dehydration) for managing LUTS in BPH. The study emphasizes the need to prioritize hydration as an integral part of the management plan for individuals with BPH at a higher risk of experiencing OH [40]. The consensus panel's recommendations for screening, diagnosing, and treating neurogenic OH and associated supine hypertension emphasize the importance of adequate hydration [41]. This collective body of evidence supports that proper hydration is a cornerstone in managing OH, regardless of the underlying cause.

The gradual change of posture-slow rising from a sitting or lying position: Studies have shown the importance of gradual postural changes in managing OH, with slow rising from a seated position strongly correlated with a lower likelihood of developing OH, particularly in older individuals. A study highlighted the benefits of adopting such a cautious approach to allow the cardiovascular system to adapt and minimize the drop in BP [42]. According to one study, slow, deliberate movements during posture change in older individuals who have experienced OH can counteract the initial BP drop within 15 seconds [43].

Avoiding prolonged standing or sitting to minimize orthostatic stress: Delayed OH, an insidious decrease in BP resulting from extended periods in an upright posture, can cause a gradual accumulation of blood in the lower limbs, resulting in dizziness [44]. Prolonged sitting also correlates with a detrimental cardio-metabolic profile, such as obesity and type 2 diabetes, which can alter the volume of blood and the elasticity of vessels [45]. Additionally, it can adversely impact endothelial function, a critical determinant of vascular tone and BP regulation [46]. Finally, the entwinement of diabetes and cardiovascular disease with extended periods of sitting can potentially exacerbate OH due to induced alterations in blood vessel function and nerve damage [47]. Interventions to truncate prolonged sitting and standing (such as adopting sit-stand desks or incorporating intermittent breaks from sitting) offer a potential mitigation strategy. However, further studies are required to analyze these complex relationships comprehensively.

Regular exercise to improve cardiovascular fitness and autonomic function: While it is logical to recommend exercise for individuals who struggle with standing tolerance, the extent to which they should exercise is uncertain. Although it may be instinctive to assume that leg resistance training can improve muscle tone and reduce venous pooling, studies have shown no significant difference in orthostatic tolerance between swimming and running training [48]. Furthermore, the findings of a study indicate that engaging in 3 hours of daily aerobic training can establish a new cardiovascular functioning set point, which may be disadvantageous for orthostatic tolerance. So, where do aerobic fitness and OH intersect? That is the gap where we need to shift attention. Regular exercise might be the key to managing OH, but to what extent is still unclear. More studies are required in order to understand better the long-term benefits exercise could offer by improving orthostatic tolerance and enhancing overall cardiovascular health [48-52].

Leg exercises and muscle contractions before standing up: Leg exercises and muscle contractions can effectively mitigate OH symptoms. The skeletal muscle pump is a mechanism by which the contraction of leg muscles helps return blood to the heart, mitigating the buildup of blood in the legs on standing. Contracting the leg muscles has also been found to minimize the decline in cerebral blood flow when changing posture. Specific exercises include calf raises, leg squats, or even tensing of leg muscles. Practicing these exercises could help reduce the incidence and severity of OH [50,53].

Wearing compression stockings and abdominal binders to enhance venous return: Compression stockings can be beneficial in managing OH. They apply pressure to the lower legs, reducing blood pooling and preventing sudden BP drops [54]. However, it is worth noting that solely compressing the legs may not be as effective as compressing the abdomen because the calf and thigh veins have a relatively small capacity compared to the splanchnic veins, constituting a significant portion of the total blood volume. Therefore, emphasizing abdominal compression as the primary strategy to reduce venous capacitance should be considered, with high-pressure compression stockings (20-30 mmHg) for additional compression to optimize outcomes [55].

Implementing tilt-table training under medical supervision for severe cases to prevent OH: Tilt-table training under medical supervision can be a viable therapeutic approach for more severe cases of OH. This strategy involves the patient being slowly tilted to an upright position (usually up to 60-80 degrees) on a table to simulate standing. This treatment encourages the body's cardiovascular system to adapt to the upright posture and enhance tolerance, which could alleviate OH symptoms over time [56].

Pharmacological Interventions

Modification of the dosage or administration schedule; initiation of treatment with a low dose with a gradual titration: As discussed earlier, A1AAs tend to relax vascular SM, resulting in systemic vasodilation and reduced BP, which can precipitate OH, especially in older individuals [57]. A widely adopted strategy is initiating treatment at a low dose to minimize these adverse effects, subsequently titrating the dose upward gradually - the principle often referred to as the 'start low, go slow' principle [58]. Furthermore, a significant reduction in the first-dose phenomenon - a sudden and severe BP drop when transitioning from a reclined to an upright position after taking an A1AA for the first time - occurred when A1AAs were titrated and given before bedtime [59]. A recent study demonstrated that initiating treatment with a lower dose of silodosin (another A1AA) was as effective as the usual dose in improving urinary symptoms with less OH in older individuals with BPH [26]. For example, alfuzosin and tamsulosin are safe and efficacious therapy options for short-term and long-term use in BPH, respectively [60].

ER formulations provide a more sustained effect with lower peak plasma levels: ER formulations offer significant advantages, particularly for the management of chronic conditions. This advanced pharmaceutical design enables a gradual, controlled, and sustained release of the active drug substance into the systemic circulation, resulting in more stable and constant therapeutic levels [61]. Additionally, ER formulations maintain steady plasma drug concentrations over time, reducing fluctuations between the maximum (Cmax) and the minimum (Cmin) concentrations that typically occur with immediate-release (IR) formulations. By preventing drug levels from peaking too high, ER formulations can reduce the risk of dose-dependent adverse events, which are more likely to occur when the drug concentration in the body reaches its peak [62].

The therapeutic profile of alfuzosin and tamsulosin has been improved, and side effects have been minimized through the development of ER formulations. Alfuzosin ER, for example, has been shown in a study to provide comparable efficacy to the IR version but with fewer reported side effects related to peak plasma drug concentrations, including better cardiovascular safety [63]. Similarly, tamsulosin ER has been associated with a more stable drug release profile. Men with cardiovascular comorbidities taking antihypertensive medications, which increase the likelihood of symptomatic hypotensive episodes, may benefit most from tamsulosin ER formulations [64]. These findings highlight the potential advantages of using ER formulations, particularly in elderly patients.

Referral to a specialist in refractory cases: Refractory OH is a condition that can lead to falls and impact a patient's QoL. OH can present with varying severity and associated morbidity, so an accurate diagnosis of the condition and its underlying etiology is crucial. As it is multifactorial and involves impaired autonomic reflexes, decreased blood volume, medications, and underlying diseases, a specialist referral may be necessary for advanced diagnostic procedures such as autonomic testing, neuroimaging, and detailed medication review [65-67]. These procedures can help determine the root cause of refractory OH and guide further management.

Specialists can provide access to advanced therapeutic options for managing refractory OH such as medications like midodrine and droxidopa. In some cases, novel interventions such as transcutaneous electrical nerve stimulation (TENS) or physical counter-maneuvers may be recommended. Referral to a specialist can provide an in-depth assessment and access to advanced treatment options, potentially leading to improved symptom control and better QoL [67].

## Conclusions

In conclusion, OH poses a significant concern for patients with BPH taking A1AAs. The incidence of this condition varies, influenced by factors such as the specific medication, dosage, treatment duration, and patient characteristics. Vigilance is key for healthcare professionals, who must assess risk factors and monitor for OH during BPH treatment. Considering age, comorbidities, medications, and treatment goals, individualized care is essential. Collaboration among healthcare providers, pharmacists, and patients is crucial to manage and prevent OH in BPH patients on A1AAs effectively.

However, there is still much to uncover about the underlying mechanisms, risk factors, and optimal management strategies for OH in this population. Further research is imperative to fill these knowledge gaps, enabling improved patient outcomes and enhancing the QoL. By addressing these challenges head-on, healthcare professionals can ensure safer and more successful BPH treatment with A1AAs, providing patients with the care they deserve.
